# Bilateral abducens nerve palsy in an infant case of fulminant acute disseminated encephalomyelitis: a case report

**DOI:** 10.1186/s12886-016-0365-3

**Published:** 2016-10-26

**Authors:** Zhiliang Yang, Guilian Sun

**Affiliations:** Department of Pediatrics, The First Affiliated Hospital of China Medical University, No. 155 Nanjing North Street, Heping District, Shenyang, 110001 Liaoning China

**Keywords:** Fulminant acute disseminated encephalomyelitis, Abducens nerve palsy, Case report, Infant

## Abstract

**Background:**

Sixth (abducens) nerve palsy (ANP) is far less frequent in children and has not been reported as a sign of acute disseminated encephalomyelitis (ADEM). We present an infant case of ADEM with bilateral abducens nerve palsy (BANP).

**Case presentation:**

We report one case of BANP in a 15-month-old boy of fulminant ADEM. The patient underwent physical examinations and brain MRI scan three times during about six months follow-up. The patient had BANP and developmental regression when he regained consciousness from a coma, and the signs had persisted for 6-months.

**Conclusions:**

BANP can be a symptom of ADEM.

## Background

Acute disseminated encephalomyelitis (ADEM) is an autoimmune inflammatory disorder of the central nervous system. The etiopathogenesis is thought to be immune-mediated. It mostly follows an antecedent infection and, rarely, an immunization [1]. The average age of onset is between 6 and 8 years and the disease is much less common in children younger than 2 years [2]. Clinical features are manifold and not pathognomonic.

Sixth (abducens) nerve palsy (ANP) is far less frequent in children. Common causes include trauma, raised intracranial pressure, and neoplasms [3]. Here, we report a rare case of ADEM in a 15-month-old boy who fell into a coma after a seizure. He had bilateral abducens nerve palsy (BANP) and developmental regression when he regained consciousness. To our knowledge, no cases of ADEM with BANP have been reported.

## Case presentation

A 15-month-old normal developmental Chinese boy was brought to our hospital with the chief complaints of having a fever for 3 days in the morning, and then one episode of seizure occurred in the afternoon. After the seizure stopped, he fell into a coma. He was evaluated and the Glasgow coma score (GCS) was 3/15 (E1V1M1). On examination, his pupils were small, the pharyngeal reflex, the pupillary, corneal, and orbital reflexes were absent. No meningeal sign was present. He had been given with intravenous antibiotic (ceftriaxone) at the clinic before hospitalization. He was first diagnosed with encephalitis.

**Fig. 1:**
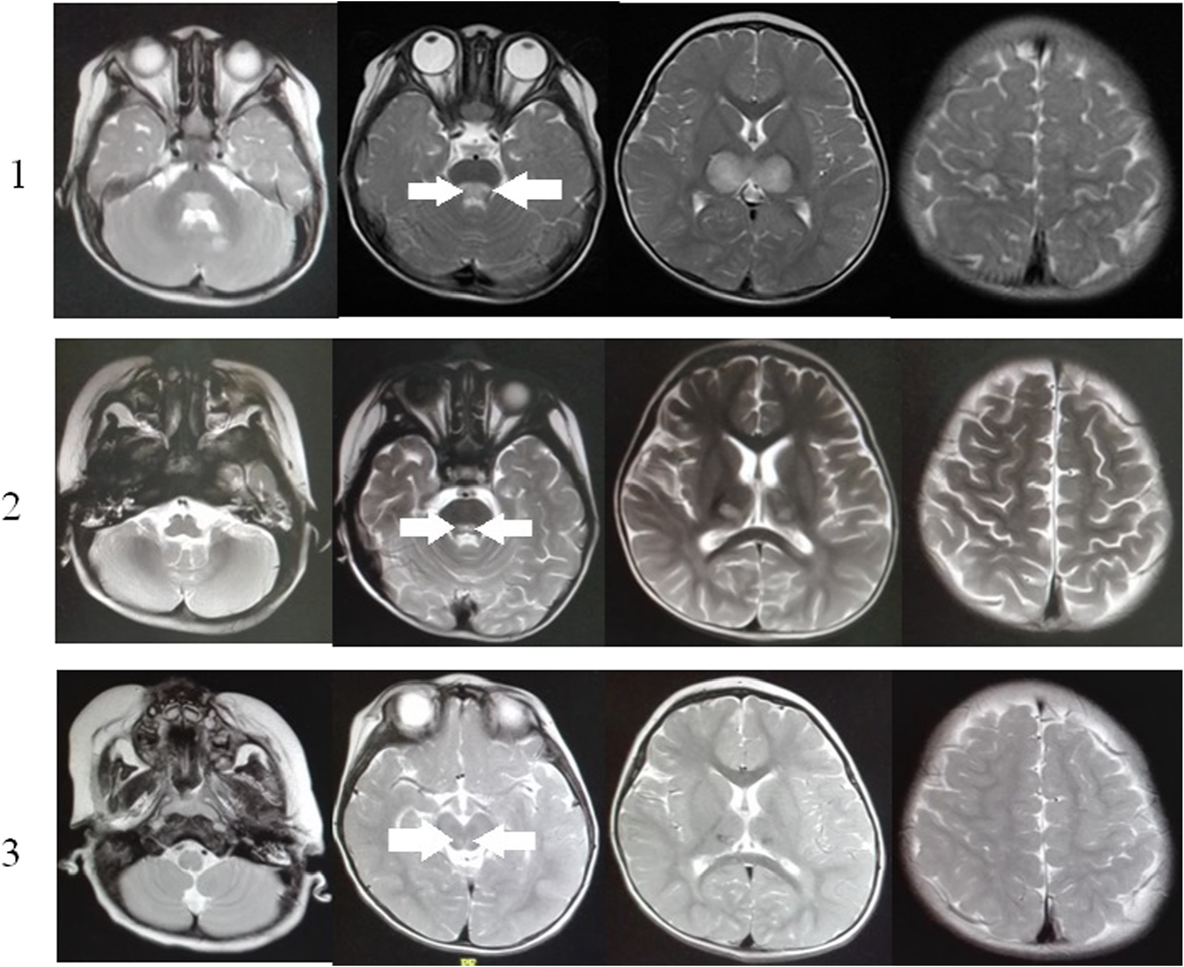
The brain T2-weighted MRI images. The leisions in the dorsal part of pons (*arrows*) were considered to cause the bilateral abducens palsy. *1*: T2-weighted MRI image showing areas of hyperintensity involving the *left* cerebellar hemisphere, the dorsal part of pons, thalamus, and *right* frontal lobe. *2*: T2-weighted MRI image after 15 days showing areas of hyperintensity that still involved the dorsal part of pons and thalamus, but not the *left* cerebellar hemisphere and *right* frontal lobe. *3*: T2-weighted MRI image after 6 months showing areas of hypointensity involving the dorsal part of pons and thalamus

A brain MRI scan was also performed urgently. The MRI images showed inhomogeneous areas of increased signal in T2-weighted images in the dorsal part of pons, midbrain, medulla, bilateral thalamus, and right frontal cortex and left cerebellar hemispheres (Fig. 1-1). An electroencephalogram revealed generalized cerebral dysfunction with no definitive irritable foci. Initial investigations revealed a white blood count of 3.4 × 10^9^/L (4-10 × 10^9^/L), hemoglobin of 125 g/L (120–140 g/L), platelet count of 180 × 10^9^/L (10-30 × 10^9^/L), C-reactive protein of 16.7 mg/dL (0–8 mg/dL), ceruloplasmin of 260 mg/L (220–330 mg/L), and lactic acid level of 7.0 mg/L (3–10 mg/dL). Fundoscopy was normal and the opening pressure on lumbar puncture was 55mmH_2_O. Lumbar puncture revealed that the cerebral spinal fluid (CSF) cell count was 3 × 10^6^/L cells (67 % lymphocytes, 33 % neutrophils), protein was 604 mg/L, and glucose was 3.4 mmol/L (capillary blood glucose was 4.1 mmol/L), and chloride was 114 mmol/L. Tests of immunoglobin M for coxsackie virus, herpes virus, cytomegalovirus, toxoplasmosis gondii, rubella virus, adenovirus, respiratory syncytial virus, and *Mycoplasma pneumoniae* were negative. The diagnosis of ADEM was considered.

He was started on intravenous methylprednisolone (250 mg, about 25 mg/kg per day) pulse therapy for 3 days and intravenous immunoglobin (IVIG; 5 g per day, about 400 mg/kg per day) for 5 days. Supportive therapies such as nasal feeding and neurotrophic drugs were applied. Hypodermic injection of interferon-α1b was also applied. The methylprednisolone pulse therapy was followed with intravenous methylprednisolone 20 mg/day (about 2 mg/kg per day) because he was unable to take prednisone orally. He showed a good response. On day 3 after starting methylprednisolone pulse therapy and IVIG, he cried and opened his eyes when a subcutaneous injection was given. The GCS was 7/15 (E4V2M1). On day 5, the GCS was 10/15 (E4V2M4). His eyeballs could move up and down, but not left and right following a light, without the presence of nystagmus, BANP was suspected. He could follow what his parents said to him and looked in the direction of their voices. however, he was too young to answer some questions. A neurological examination revealed that he could not control his head well.

On day 20, the power in his upper and lower limbs were about IV/V, and he could control his head better, turn over, and crawl a little, but he could not sit or stand unsupported. The MRI imaging of brain and spinal cord were checked. Increased signal in T2-weighted images (Fig. 1-2) was still observed in the bilateral thalamus, the dorsal part of pons, midbrain still, and no abnormal signals were seen in the spinal cord.

The boy was discharged on day 22. At the time of discharge, he was clear consciousness, with a GCS of 11/15 (E4 V2 M5). Oral prednisone 5 mg was given twice daily for about one month, then tapered to 5 mg once daily for about one month and finally to 5 mg every two days.

Three months after the onset, he could sit without support but still could not walk without assistance. He also showed ataxia when he tried to grab something with his hands. Both eyes showed esotropia and could not abduce horizontally, which was compatible with the lesion in the the dorsal part of pons in images, and acquired central BANP was confirmed. At the 6-month follow-up, the MRI images (Fig. 1-3) showed hypointensity involving the the dorsal part of pons and thalamus, and the child’s movement disablement and fixed esotropia still existed.

## Discussion

The annual incidence of ADEM is presumed to be 0.8/100,000 persons. ADEM may begin abruptly, acutely, or over a period of a few days, typically within 1 to 2 weeks following an antigenic challenge. Neurological symptoms typically evolve over several days in ADEM, and alterations in consciousness may range from mild irritability or somnolence to prominent behavioral change or coma [4]. There are no specific biomarkers currently available to diagnose ADEM, hence, diagnosis is made after excluding clinical and laboratory findings, and relies on suggestive neuroradiological features of ADEM.

To exclude hereditary metabolic diseases, blood ceruloplasmin and lactic acid levels were tested and were normal. CSF is usually normal, and a mild elevation of protein with lymphocytic pleocytosis may sometimes be found. Our case had mild elevation of CSF protein without abnormal cells. Some studies have suggested that bilateral thalamic lesions on MRI images may be diagnostic for ADEM [5–7]. In our case, the brain MRI showed lesions in the pons, midbrain, medulla, bilateral thalamus, right frontal cortex, and left cerebellar hemisphere. The lesions in the bilateral thalamus were larger than 2 cm and tumefactive, which were the most important features for the diagnosis in our case. The diagnosis of our patient was based on his clinical presentation with fever, altered level of consciousness, motor deficits, and brain imaging findings, according to the revised ADEM definitions in 2013 [8].

Signs of cranial nerve involvement are present in 20–37 % of patient with ADEM [9, 10], but ANP has not been reported. In our case, the child’s eyeballs were able to move up and down, but not left and right following a light after he regained consciousness. After about 2 months, esotropia was observed and his eyeballs could not abduce horizontally. The lesions in the bilateral thalamus and pons still existed at the 6-month follow-up. The leisions in the dorsal part of pons are compatible with the locations of abducens nucleus. The lesions in the abducens nucleus were considered, acquired central BANP could be confirmed.

## Conclusions

We report the case of an infant with fulminant ADEM that showed fixed esotropia and marked developmental regression during 6 months. The BANP and developmental regression can be symptoms of ADEM in infants.

## Abbreviations

ADEM: Acute disseminated encephalomyelitis
ANP: Abducens nerve palsy
BANP: Bilateral abducens nerve palsy
CSF: Cerebral spinal fluid
GCS: Glasgow coma score
IVIG: Intravenous immunoglobin
